# Editorial: Current concepts of cellular and biological drugs to modulate regulatory T cell activity in the clinic, volume II

**DOI:** 10.3389/fimmu.2023.1221904

**Published:** 2023-06-13

**Authors:** Valentin Wenger, Robert Zeiser

**Affiliations:** ^1^ Department of Medicine I, Medical Center - University of Freiburg, Faculty of Medicine, University of Freiburg, Freiburg, Germany; ^2^ Signaling Research Centre for Biological Signalling Studies (BIOSS) Freiburg and CIBSS – Centre for Integrative Biological Signalling Studies, University of Freiburg, Freiburg, Germany; ^3^ Comprehensive Cancer Center Freiburg (CCCF), Medical Center - University of Freiburg, Faculty of Medicine, University of Freiburg, Freiburg, Germany

**Keywords:** regulatory T (Treg) cells, graft-versus host disease, CD73, GARP, anti-tumor immune response, autoimmune diseases

Regulatory T cells (Tregs) represent a group of specialized CD4^+^ T cells that play a pivotal role in the maintenance of immune homeostasis and tolerance. In several diseases, however, the presence of Tregs interferes with a desirable effect to combat disease. This is specifically important in malignancy where Tregs have been shown to reduce the anti-tumor effects of immune checkpoint inhibitor (ICI) such as anti-PD-(L)1 (programmed cell death 1 (ligand) protein 1 or anti-CTLA-4 (cytotoxic T-lymphocyte-associated protein 4) and thereby promote secondary resistance. On the other hand, a lack of functional Tregs may enhance autoimmune diseases, such as rheumatoid arthritis, systemic lupus erythematosus, severe allergy, and allogeneic immune responses such as allograft rejection or graft-versus-host disease (GvHD).

In this editorial, the authors cover recent advances on Tregs including the role of glycoprotein A repetitions predominant (GARP), ectonucleotidases CD39 and CD73, as well as nanodrugs targeting T cells.

Tregs exert immunosuppression *via* multiple mechanisms including suppression of antigen-presenting cells (APCs), secretion of anti-inflammatory cytokines, reprogramming of metabolic functions, and direct cytotoxic effect.

Eschborn et al. elucidated on the role of the Transforming Growth Factor β (TGFβ)- activating transmembrane GARP. They demonstrated that depletion of GARP^+^ Tregs in food allergy results in increased gastrointestinal inflammation (1). Interestingly, Krebs et al. reported that patients with melanoma who did not respond to ICI had a longer survival if they expressed low frequency of GARP^+^ Tregs (2). In addition, Satoh et al. have described that the anti-GARP antibody DS-1055a is an effective treatment in a humanized mouse model of colorectal cancer (3). On the other hand, Wang et al. highlighted that GARP^+^ Tregs attenuate GvHD (4).

Another mechanism of action for Tregs’ suppressive functions is their ectonucleotide enzymes CD39 and CD73 that convert ATP to ADP/AMP and adenosine conversely, conferring local and short-lasting immune suppression. Allard et al. reported that CD73 has been upregulated in several tumor entities, such as ovarian, colon, breast cancer, and leukemia, and concluded that CD73 expression increases the risk for metastasis and chemoresistance (5). In addition, Allard et al. also reported that, by anti-CD73 monoclonal antibody treatment, effector T cells (Teff) and APCs potentiate Th1 response and IFNγ production due to the synergistic effect of CD73 and PD1 blockage (6). In a head and neck cancer model, Deng et al. demonstrated that CD73-blockade reduced tumor growth and reversed exhausted CD4^+^ and CD8^+^ T-cell phenotype (7). The role of CD73 in GvHD and anti-tumor immune responses has been reported in the past. Tsukamoto et al. revealed that pharmacological inhibition of CD73 resulted in enhanced graft-versus-tumor response (8).

A major hurdle for targeting Tregs in autoimmune diseases and cancer remains the difficulty to specifically target these cells. Therefore, the use of nanodrug carriers (NCs) has shown to be a promising approach, thereby maximizing on-target while minimizing any off-target effects. NCs targeting Teff and Tregs are an especially useful tool as they can be used for depletion/reprogramming of immunosuppressive cells, and APCs mediated T- cell activation or enhancement of immunogenic cell death. One such approach is the use of protein-based nanodrug (Nab-paclitaxel) that exhibited rapid tumor response for patients with metastatic breast cancer (9). Other groups demonstrated that polymer-based doxorubicin (Doxil) depleted myeloid-derived suppressor cells and increased cancer cell susceptibility to granzyme B while reducing cardiotoxicity (10–12).

In summary, this editorial provides an overview over several novel interventions employing a variety of therapeutic moieties that result in therapeutic up- or down-modulation of Treg function. Future studies will reveal whether some of these modalities are effective and safe enough to become part of our treatment armamentarium to combat autoimmunity and cancer.

## Author contributions

All authors listed have made a substantial, direct, and intellectual contribution to the work and approved it for publication.
